# *LYRM1*, a Gene that Promotes Proliferation and Inhibits Apoptosis during Heart Development 

**DOI:** 10.3390/molecules15106974

**Published:** 2010-10-11

**Authors:** Chun Zhu, Yao-Qiu Liu, Fu-Kun Chen, De-Liang Hu, Zhang-Bin Yu, Ling-Mei Qian

**Affiliations:** 1 Department of Pediatrics, Nanjing Maternal and Child Health Hospital of Nanjing Medical University, No.123 Tianfei Road, Nanjing 210004, China; 2 Department of Cardiology, The First Affiliated Hospital of Nanjing Medical University, No.300 Guangzhou Road, Nanjing 210029, China

**Keywords:** CHD, *LYRM1*, P19 cells, heart development

## Abstract

Congenital heart disease (CHD) is the most common type of birth defect, but its underlying molecular mechanisms remain unidentified. Previous studies determined that *Homo sapiens* LYR motif containing 1 (*LYRM1*) is a novel nucleoprotein expressed at the highest level in adipose tissue and in high levels in heart tissue. The *LYRM1* gene may play an important role in the development of the human heart. This study was designed to identify the biological characteristics of the *LYRM1* gene in heart development. On the basis of expression-specific differentiation markers identified with quantitative real-time RT-PCR and the morphology of *LYRM1*-overexpressing cells during differentiation, ectopic expression was not found to significantly affect differentiation of P19 cells into cardiomyocytes. MTT assays and cell cycle analysis showed that *LYRM1* dramatically increases the proliferation of P19 cells. Furthermore, data from annexin V-FITC binding and caspase-3 activity revealed that *LYRM1* can inhibit the apoptosis of P19 cells. Our data suggest that *LYRM1* might have the potential to modulate cell growth, apoptosis, and heart development.

## 1. Introduction

Congenital heart disease (CHD) is the most common type of human birth defect; its incidence is 6–8 per 1,000 live births [1]. In China, there are more than 4 million untreated CHD patients [2]. Although the mortality of children with CHD has decreased because of improved surgical treatments, not all CHD patients survive to adulthood. CHD is a multifactorial disease, with environmental and genetic factors playing important roles [3]. According to existing epidemiologic studies, many genes are involved in the regulation of heart development, yet its molecular etiology is poorly understood. 

There are at least eight LYR motif (*LYRM*) proteins in *Homo sapiens*. *LYRM1*, a novel gene, could play a role in cell growth and apoptosis. Previous studies have shown that besides showing the highest expression in adipose tissue, *LYRM1* is also abundantly expressed in human heart tissue [4]. Therefore, this gene was selected for further analysis of heart development. In this study, we initially examined the effect of *LYRM1* on cell differentiation. We found that overexpression of *LYRM1* did not affect the differentiation of P19 cells into cardiomyocytes. Furthermore, we examined the effect of *LYRM1* on cell proliferation and apoptosis and found that it dramatically increased the growth rate of the P19 cells and protected P19 cells from apoptosis. 

## 2. Results and Discussion

### 2.1. Effects of LYRM1 on cell differentiation

**Figure 1:**
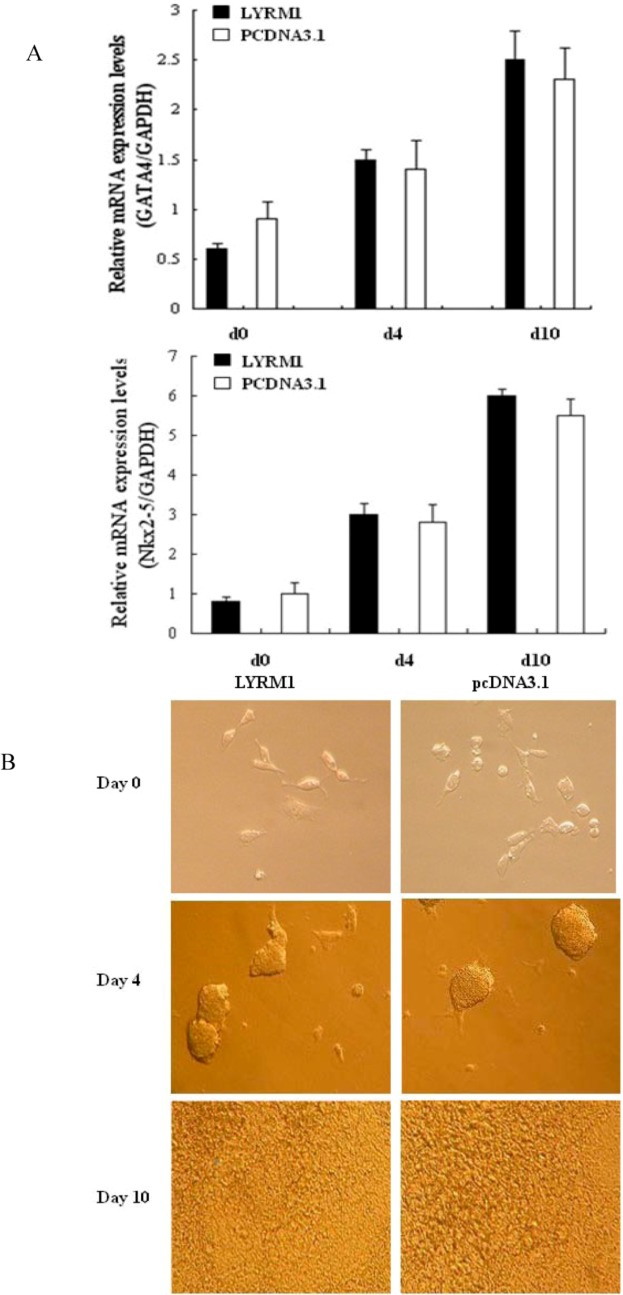
Effect of *LYRM1* on cell differentiation. (A) Expression of specific differentiation markers as determined by quantitative real-time RT-PCR at various time points during the stimulation of differentiation (days 0, 4, and 10). No difference in the expression levels of these markers was found between cells with or without exogenous expression of *LYRM1* (n = 6; *P* < 0.05). (B) Morphology of P19 cells during differentiation (days 0, 4, and 10). P19 cells were transfected with pcDNA3.1Myc/HisB-LYRM1 or an empty vector and stimulated to differentiate over 10 days. This experiment is representative of three independent assays.

In order to assess whether *LYRM1* affects the differentiation of P19 cells into cardiomyocytes, we established P19 cells that stably overexpressed *LYRM1* and assayed the ability of these cells to differentiate into cardiomyocytes. The control was P19 cells transfected with pcDNA 3.1/HisB. No significant difference in morphology between P19 cell lines overexpressing the *LYRM1* gene on days 0, 4, and 10 of differentiation and the control cells was found (Figure 1A. In a subsequent study, the expression of cardiomyogenesis-specific markers was detected during the differentiation of P19 cells. The molecular markers included *GATA4* and *Nkx2-5*. Results from real-time qRT-PCR showed that there was no difference in the mRNA expression of these marker genes between *LYRM1*-overexpressing cells and controls at the same time points (days 0, 4, and 10) (Figure 1B).

### 2.2. Effect of LYRM1 on cell proliferation

**Figure 2:**
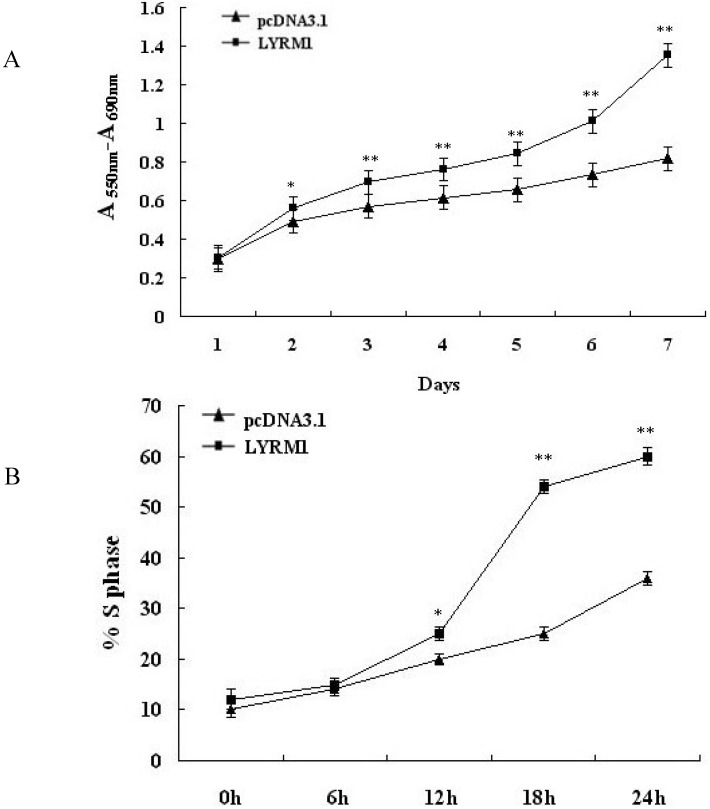
Effect of *LYRM1* on cell proliferation. The growth of cells was detected by MTT assays (A) and cell cycle analysis (B). Ectopic expression of *LYRM1* increases P19 cells proliferation. The figure represents one of three independent experiments (^*^P = 0.001, ^**^P < 0.001).

Cell proliferation rate was analyzed with MTT assays every day for 7 days. P19 cells stably transfected with the pcDNA3.1Myc/HisB vector served as controls. Overexpression of *LYRM1* resulted in a much higher growth rate compared with control cells ( 2A, ^*^P = 0.001, ^**^P < 0.001). Cell cycle analysis of stably transfected cells was used to further evaluate the effect of *LYRM1* on cell proliferation. Data from flow cytometry assays showed that the percentage of P19 cells overexpressing *LYRM1* in the S-phase compartment increased from 12 h after serum stimulation compared with control cells (Figure 2B, ^**^P < 0.001).

### 2.3. Effect of LYRM1 on cell apoptosis

**Figure 3:**
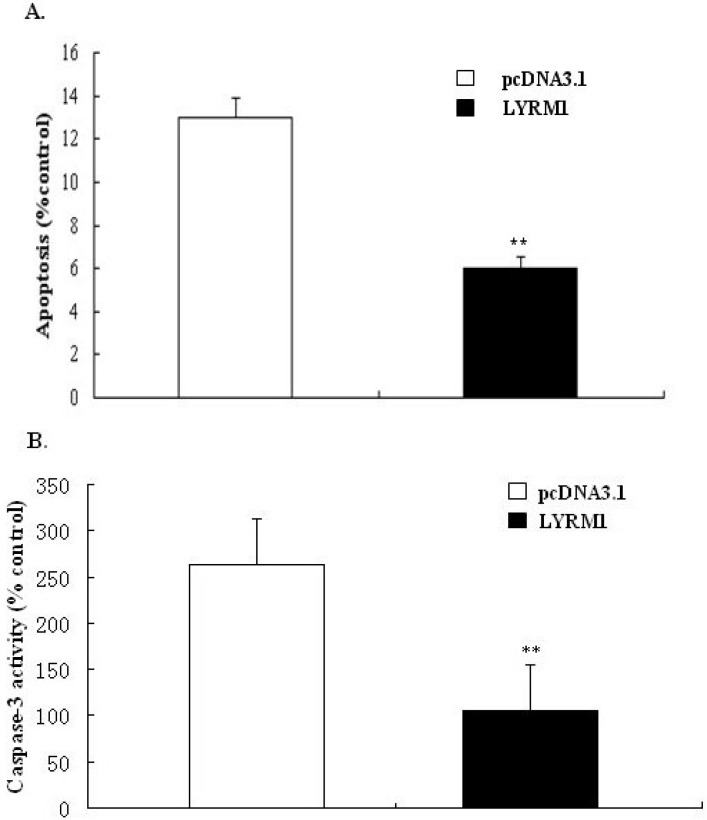
Effect of *LYRM1* on cell apoptosis. Apoptosis was assayed by detecting binding of annexin V-FITC (A) and caspase-3 activity (B). Data from both assays showed that *LYRM1* inhibits serum deprivation-induced apoptosis (n = 6; ^**^P < 0.001)

To test the effect of *LYRM1* on cell apoptosis, we cultured P19 cells in the absence of FBS for 24 h. The number of phosphatidylserine-positive cells was quantified using annexin V staining and by measuring caspase-3 activity; these assays were used to measure apoptosis. Our results suggest that *LYRM1* protects against serum deprivation-induced apoptosis (Figure 3). 

### 2.4. Discussion

Chromosomal and Mendelian syndromes account for approximately 20% of human CHD [5]. Although many genes have been found to be important for cardiac development in animal studies, only a few genes related to CHD in humans have been identified [6]. The results of multi-tissue expression analysis support a role for *LYRM1* in human heart development. However, its function in heart development is currently unknown [4]. 

The heart is the first organ to form in the embryo, and a complex series of morphogenetic interactions involving cells from several embryonic origins is involved in its development [7]. Embryonic-fetal heart growth depends on the balance between proliferation and apoptosis of myocytes [8]. Previous reports have shown that P19 cells are a powerful model for studying the regulation of myocardial differentiation at the molecular and functional levels [9,10]. Therefore, in the present study, we used the P19 cell line as a research model. By establishing a stably transfected P19 line that overexpressed *LYRM1*, we found that *LYRM1* does not affect the differentiation of P19 cells into cardiomyocytes, as shown by the morphology and expression of cardiomyogenesis-specific molecular markers. GATA4 is a cardiac-specific member of the GATA family of zinc finger transcription factors, and it is essential for cardiac differentiation of embryonal carcinoma cell lines [11,12,13]. Nkx2.5, a member of the NK2 class homeodomain protein, is required for early heart development [14,15,16]. Moreover, early studies found that GATA4 and Nkx2.5 interact physically and synergistically to activate cardiac transcription, suggesting that they play vital roles in mesoderm formation and specification of cardiac progenitors [17,18,19]. The MTT assays revealed that P19 cells overexpressing *LYRM1* grew faster than the controls, and cell cycle analysis indicated a dramatic increase in the percentage of *LYRM1*-overexpressing P19 cells in the S phase. These data showed that *LYRM1* increases the proliferation of P19 cells. Furthermore, the results from the analysis of annexin V-FITC staining and caspase-3 activity indicate that *LYRM1* can prevent apoptosis induced by serum removal. 

## 3. Experimental

### 3.1. Cell culture and differentiation of P19 cells

P19 cells were obtained from the American Type Culture Collection (ATCC, Manassas, VA, USA). The cells were cultured in α-modified Eagle’s medium (α-MEM; Gibco BRL, Grand Island, NY, USA) containing 10% fetal bovine serum (FBS, Gibco BRL), 100 U/mL penicillin, and 100 μg/mL streptomycin at 37 °C in 5% CO_2_. In order to induce cardiac differentiation, embryoid body (EB) formation was induced by plating 1 × 10^6^ P19 cells on 10-cm bacterial dishes in 15 mL of α-MEM supplemented with 1% dimethyl sulfoxide (DMSO, Sigma, St. Louis, MO, USA), 10% FBS, 100 U/mL penicillin, and 100 μg/mL streptomycin for 96 h. The formed EBs were transferred to 6-cm bacterial dishes and cultured in α-MEM with 10% FBS for an additional 4 or 6 days. The morphologic changes in the P19 cells were examined and photographed using an inverted microscope (Nikon, Japan).

### 3.2. Quantitative real-time RT-PCR

Total RNA was extracted from the P19 cells with Trizol reagent (Invitrogen, Carlsbad, CA, USA). Then, cDNA was synthesized from 1 μg of total RNA using an AMV Reverse Transcriptase Kit (Promega A3500; Promega, Madison, WI, USA). Real-time PCR was performed using the SYBR Green method in an Applied Biosystems 7300 Sequence Detection System (ABI 7300 SDS; Foster City, CA, USA) following the manufacturer’s protocols. The PCR conditions included a denaturation step (95 °C for 10 min), followed by amplification and quantification repeated 40 times (95 °C for 15 s and 60 °C for 1 min). Gene expression was measured in triplicate. The relative gene expression levels were quantified based on the Ct and normalized to a reference gene, *GAPDH*. The sequences of the primers used are shown in Table 1.

**Table 1:** Sequences of the primers used in this study.

Gene name	Product size	Reverse and forward primers (5'-3')
*GATA4*	136bp	F:5'-CCTGCGGCCTCTACATGA-3'
		R:5'-AGGGTCTCACCAGCAGGA-3'
*Nkx2-5*	222bp	F:5'-CCTGCGGCCTCTACATGA-3'
		R:5'-AGGGTCTCACCAGCAGGA-3'
*GAPDH*	237bp	F:5'-TTCACCACCATGGAGAAGGC-3'
		R:5'-GGCATGGACTGTGGTCATGA-3'

### 3.3. Establishment of a stable cell line overexpressing LYRM1

The coding sequence of *LYRM1* was subcloned into the *Bam*HI and the *Xho*I sites of the pcDNA3.1Myc/HisB vector to generate a plasmid expressing the LYRM1-6×His fusion protein. Expression vectors carrying the *LYRM1* coding sequence, or empty vectors, were transfected into P19 cells. Two days after transfection, neomycin (G418, Roche, Basel, Switzerland) was added to the medium (600 μg/mL) to select for transfected P19 cells. After 2 weeks, individual colonies were isolated, propagated, and the LYRM1-6×His fusion protein was confirmed by western blotting. The anti-6×His antibody was obtained from Clontech. Colonies expressing the highest levels of *LYRM1* were selected for further studies.

### 3.4. MTT assay

Cells were seeded in 96-well plates at a density of 2 × 10^2^ cells/well and maintained in media supplemented with 10% FBS and G418 250 μg/mL. The growth of the cells was analyzed for 7 consecutive days by 3-(4,5-dimethylthiazol-2-yl)-2,5-diphenyltetrazolium bromide (MTT) assays, using a Cell Proliferation MTT Kit (Roche Diagnostics). Absorbance was measured at 560 nm and 660 nm, as recommended by the manufacturer.

### 3.5. Cell cycle assay

Before analysis, the cells were starved in serum-free α-MEM for 24 h to synchronize the cell cycle. Cells were incubated with α-MEM containing 10% (v/v) FBS for various times (0, 6, 12, 18, and 24 h) after serum deprivation. Cells were harvested using trypsin/EDTA, fixed in 70% ethanol, and cellular DNA was stained with 500 μL propidium iodide (PI). Cultured cells were analyzed by flow cytometry, and data were analyzed using CellQuest software.

### 3.6. Apoptosis assay

Cells were cultured in serum-free α-MEM for 24 h to induce apoptosis, and then harvested using trypsin/EDTA and washed with PBS. The cells were centrifuged and resuspended in 1 mL binding buffer and then stained with 10 μL annexin V-FITC and 10 μL PI at room for 5 min (Biovision, CA, USA). The stained cells were immediately analyzed using flow cytometry as previously described [20]. 

### 3.7. Measurement of caspase-3 activity

Caspase-3 activity was measured using a commercially available kit (KeyGen, Nanjing, China). The cells (5 × 10^6^) were lysed with 50 μL of chilled cell lysis buffer on ice for 20 min. After microcentrifugation (10,000 rpm, 1 min, 4 °C), the supernatant was used for a caspase-3 colorimetric protease assay. Next, 150 μg of protein was diluted to 50 μL with cell lysis buffer and then incubated with 5 μL substrate at 37 °C for 4 h. The samples were read at 405 nm in a microtiter plate reader.

### 3.8. Statistical analysis

The data are presented as the mean ± SD. All values were analyzed using the Student’s *t* test with the SPSS 15.0 statistical package. *P* values < 0.05 were considered statistically significant.

## 4. Conclusions

In summary, we have described several useful features of the novel gene *LYRM1* in cardiac development. However, the precise molecular function of this gene remains unknown. For example, the *in vivo* function of *LYRM1* remains to be clarified. Further studies are also required to elucidate the biological function of *LYRM1* in the differentiation of human cardiac cells.
